# Two complementary approaches to estimate an excess of mortality: The case of Switzerland 2022

**DOI:** 10.1371/journal.pone.0290160

**Published:** 2023-08-15

**Authors:** Isabella Locatelli, Valentin Rousson

**Affiliations:** Center for Primary Care and Public Health (Unisanté), University of Lausanne, Lausanne, Switzerland; University of Zurich, SWITZERLAND

## Abstract

**Objective:**

During the COVID-19 pandemic, excess mortality has generally been estimated comparing overall mortality in a given year with either past mortality levels or past mortality trends, with different results. Our objective was to illustrate and compare the two approaches using mortality data for Switzerland in 2022, the third year of the COVID-19 pandemic.

**Methods:**

Using data from the Swiss Federal Statistical Office, standardized mortality rates and life expectancies in 2022 were compared with those of the last pre-pandemic year 2019 (first approach), as well as with those that would be expected if the pre-pandemic downward trend in mortality had continued during the pandemic (second approach). The pre-pandemic trend was estimated via a Poisson log-linear model on age-specific mortality over the period 2010–19.

**Results:**

Using the first approach, we estimated in Switzerland in 2022 an excess mortality of 2.6% (95%CI: 1.0%-4.1%) for men and 2.5% (95%CI: 1.0%-4.0%) for women, while the excess mortality rose to 8.4% (95%CI: 6.9%-9.9%) for men and 6.0% (95%CI: 4.6%-7.5%) for women using the second approach. Age classes over 80 were the main responsible for the excess mortality in 2022 for both sexes using the first approach, although a significant excess mortality was also found in most age classes above 30 using the second approach. Life expectancy in 2022 has been reduced by 2.7 months for men and 2.4 months for women according to the first approach, whereas it was reduced by respectively 8.8 and 6.0 months according to the second approach.

**Conclusions:**

The excess mortality and loss of life expectancy in Switzerland in 2022 are around three times greater if the pre-pandemic trend is taken into account than if we simply compare 2022 with 2019. These two different approaches, one being more speculative and the other more factual, can also be applied simultaneously and provide complementary results. In Switzerland, such a dual-approach strategy has shown that the pre-pandemic downward trend in mortality is currently halted, while pre-pandemic mortality levels have largely been recovered by 2022.

## Introduction

Since 2020, most countries in the world have been facing the COVID-19 pandemic caused by severe acute respiratory syndrome coronavirus 2 (SARS-CoV-2). After officially killing about 5.3 million people worldwide in the first two years 2020–21, the pandemic has still generated about 1.4 million deaths and remained globally active in 2022 (COVID–Coronavirus Statistics–Worldometer (worldometers.info)). In Switzerland, the official numbers of deaths associated with COVID-19 were of 7610 in 2020, 4335 in 2021 and 1882 in 2022, according to the Federal Office of Public Health (COVID-⁠19 Suisse | Coronavirus | Dashboard (admin.ch)). However, these statistics could be misleading due to variability between countries in reporting and testing COVID-19, possible misclassification of causes of death, and because of their inherent inability to account for mortality indirectly generated by the pandemic. This is why it has been recommended to measure the impact of COVID-19 based on excess of overall mortality [1]. Some studies have estimated an excess of 15 to 18 million deaths worldwide for the period 2020–21, about three times the number of deaths officially attributed to COVID-19 [2, 3]. In Switzerland, overall mortality was studied by [4, 5] in 2020 and 2021, showing an increase of 9.2% in 2020 compared to 2019, followed by an approximate recovery in 2021, with a remaining 0.8% compared to 2019, corresponding to an excess of about 7000 deaths for the period 2020–21.

When calculating an excess mortality for a given year, the question arises as to whether this should be done relative to a given past mortality level, typically that of the last pre-pandemic year(s), in what follows the *first approach*, or relative to a predicted mortality obtained by modelling past trends, in what follows the *second approach*. Both approaches were indeed adopted to estimate an excess mortality in 2020–21. Studies [4, 5] for Switzerland were examples of the first approach. Mortality in 2020 was also compared with that of the last pre-pandemic year 2019 by [6] for the USA and by [7] for England. A comparison of mortality in 2020 with average mortality over the period 2015–19 was carried out for Italy by [8] and for 35 countries by [9], whereas similar calculations were made for Portugal by [10], based on average mortality over the 2010–19 period. The second approach has also been widely used, with various (generalized) linear models applied to selected pre-pandemic periods to predict the expected mortality in 2020 (in the absence of a pandemic), for comparison with observed mortality. For example, a piecewise linear model was applied by [11] to mortality data in Iran over the period 2015–19. The same pre-pandemic period was considered by [12] to predict, using a simple linear model, the expected number of deaths in Russia for 2020. A generalized additive model with a negative binomial distribution was applied by [13] to mortality data in England for the period 2010–19, while a Poisson model was applied by [14] to mortality data of 2016–19 in 29 high-income countries. A similar model was adopted for the USA by [15] to estimate a trend over the period 2014–2019. In Switzerland, excess mortality to 2020 was estimated by [16] by means of a Bayesian model over the period 2015–19. Finally, a combination of several spline models was adopted by [2, 3] to estimate excess mortality for a large number of countries and on a global scale.

Considering that mortality rates, adjusted for demographic changes, tended to decline in most countries prior to the pandemic, an excess mortality that takes this trend into account will generally appear more dramatic than a simple comparison with pre-pandemic levels. For example, the excess mortality in 2020 in the United States was estimated to be 16% by [6], who compared the standardized mortality rate of 2020 to that of 2019, while an estimate of 23% was obtained by [15], who compared mortality in 2020 to an estimated trend. In Switzerland, an excess mortality of 12.5% has been obtained by [16] comparing mortality in 2020 with a trend estimated by a Bayesian model, which is larger than the excess mortality of 9.2% [5], obtained by comparing 2020 with 2019. The latter authors also mentioned that the excess of about 7000 deaths estimated for the period 2020–21 by comparing both years with 2019 would become about 10’500 deaths had the authors based their calculation on a linear trend estimated over 2010–19.

Another widely adopted indicator to quantify the impact of a pandemic on mortality is life expectancy at birth [17]. The two general approaches discussed above could also be applied in this context, since the question of whether a loss of life expectancy in a given year should be calculated relative to past levels or past trends also applies here. Perhaps surprisingly, although a large literature exists in the field of life expectancy modelling and extrapolation [18–20], the impact of the COVID-19 pandemic on life expectancy in 2020 and 2021 has generally been measured by a simple comparison with pre-pandemic levels (e.g. 2019) [21, 22], i.e. using the first approach only.

Three years after the start of the pandemic, the question whether one should compare the current mortality with pre-pandemic levels or with estimated trends becomes even more important. The main goal of the present paper is to compare the two approaches both in terms of excess mortality and of life expectancy loss through an analysis of mortality in Switzerland in 2022. Another goal is to complement our previous analyses of mortality in Switzerland in 2020 and 2021 [4, 5], to provide a follow-up of the impact of COVID-19 in this country.

## Data and methods

### Standardized weekly deaths

Weekly mortality for the years 2015–22 was analyzed via Standardized Weekly Deaths (SWD). We used the numbers of deaths by 5 age classes (0–19, 20–30, 40–64, 65–79, 80+) for each of the 52 (or 53) weeks of years 2015–22 (provisional FSO data: Décès par classe d’âge, semaine et canton—29.12.2014–4.6.2023 | Tableau | Office fédéral de la statistique (admin.ch), last access June 13, 2023) and the sizes of the Swiss population as of January 1 of the same years (FSO data: Population résidante permanente selon l’âge, le sexe et la catégorie de nationalité, de 2010 à 2021–2010–2021 | Tableau | Office fédéral de la statistique (admin.ch)) grouped in the same age classes. Reference for standardization was set at January 1, 2022. The SWD of week *w* (*w* = 1,…,52 or 53) and year *y* (*y* = 2015,…,2022) standardized at January 1 of year *s* (*s* = 2022) was calculated as follows:

$$d^{w,y,s}=\sum_{k=1}^{5}\frac{D_{k}^{w,y}}{P_{k}^{y}}P_{k}^{s}.$$

In this formula $D_{k}^{w,y}$ represents the number of observed deaths for week *w* of year *y* and age class *k*, and $P_{k}^{y}\;(P_{k}^{s})$ is the population size of age class *k* as of January 1 of year *y* (*s*).

### Standardized mortality rates

Annual mortality for the years 2010–22 was analyzed by sex and one-year of age (directly) Standardized Mortality Rates (SMR) [23].

For the period 2010–21, we used the annual number of deaths, separately for men and women, as available by 1-year age class (with a last open class of 110+) by the FSO (Décès selon l’âge et le sexe, de 1970 à 2021–1970–2021 | Tableau | Office fédéral de la statistique (admin.ch)), and the size of Swiss population as of January 1, 2010–22 by sex and 1-year age class (with a last open class of 105+) also available by the FSO (Population résidante permanente selon l’âge, le sexe et la catégorie de nationalité, de 2010 à 2021–2010–2021 | Tableau | Office fédéral de la statistique (admin.ch)). The SMR for year *y* (*y* = 2010,…,2021) and sex *j* (*j* = man/woman) standardized at January 1 of year *s* (*s* = 2022) was obtained as follows:

$$m_{j}^{y,s}=\sum_{i=0}^{100+}m_{ij}^{y}\frac{P_{ij}^{s}}{P_{j}^{s}}.$$

In this formula, $m_{ij}^{y}=D_{ij}^{y}/P_{ij}^{y}$ represents the age and sex specific mortality rate for age *i* and sex *j* in year *y*, $D_{ij}^{y}$ is the number of observed deaths for age *i* and sex *j* in year *y*, $P_{ij}^{y}\;(P_{ij}^{s})$ is the population size of age *i* and sex *j* of the year *y* (*s*), and $P_{j}^{s}$ the total population size of sex *j* in the reference year: $P_{j}^{s}=\sum_{i=0}^{100+}P_{ij}^{s}$. To facilitate comparisons across all years, we excluded 1/366 of deaths for leap years (2008-2012-2016-2020). For comparison, we also calculated Crude Mortality Rates (CMR) as $m_{j}^{y}=D_{j}^{y}/P_{j}^{y}$, with $D_{j}^{y}=\sum_{i=0}^{100+}D_{ij}^{y}$ and $P_{j}^{y}=\sum_{i=0}^{100+}P_{ij}^{y}$.

For the year 2022 the number of deaths was available on a weekly basis by 5-years age classes (with a last open class of 90+) (provisional FSO data: Décès selon la classe d’âge quinquennale, le sexe, la semaine et le canton—3.1.2022–4.6.2023 | Tableau | Office fédéral de la statistique (admin.ch), last access June 13, 2023). Since 2022 is the reference year *y* = *s* = 2022, the SMR corresponds to the CMR in that year: $m_{j}^{s,s}=m_{j}^{s}=D_{j}^{s}/P_{j}^{s}$. Here, the total number of deaths $D_{j}^{s}$ was obtained by summing up the number of deaths of all weeks of 2022. By convention, the year 2022 is divided into 52 weeks, the first of which begins on January 3, 2022, and the last ends on January 1, 2023. Therefore, before aggregating the weekly deaths, we added 2/7 of the deaths in the first week and excluded 1/7 in the last. All SMRs were also calculated by 10-year age classes (with a last open class of 90+).

### Modelling trends

To model a trend for the SMRs of men and women over the pre-pandemic years, we fitted a Poisson log-linear model to the $D_{ij}^{y}$ of years *y* = 2010–19, separately for each sex *j*, with (1-year of) age *i* as categorical factor, year *y* as covariate (to estimate a linear trend on the log scale), interactions between age and year (to allow a possibly different trend at each age), and an offset to account for the different population sizes (Chapter 2.4 of [24]). For years *y* = 2010−22, predicted sex- and age-specific mortality rates ${\hat{m}}_{ij}^{y}$ based on the estimated trend, as well as their variances on the log scale $V[log\;{\hat{m}}_{ij}^{y}]$ (*i* = 0,…,100 and *j* = man/woman) resulting from the model, were combined to obtain predicted SMRs and their approximated variances (via the delta method):

$${\hat{m}}_{j}^{y,s}=\sum_{i=0}^{100+}{\hat{m}}_{ij}^{y}\frac{P_{ij}^{s}}{P_{j}^{s}}$$

$$V[\log\;{\hat{m}}_{j}^{y,s}]=\frac{1}{{\left({\hat{m}}_{j}^{y,s}\right)}^{2}}\sum_{i=0}^{100+}{\left({\hat{m}}_{ij}^{y}\frac{P_{ij}^{s}}{P_{j}^{s}}\right)}^{2}V[log\;{\hat{m}}_{ij}^{y}].$$

Similar calculations were done to obtain predicted SMRs in specific age classes.

### Excess mortality

An Excess Mortality (EM) between an SMR observed one given year *y* and that from a past year *z* (for example *y* = 2022 and *z* = 2019) was defined for each sex *j* by the relative change of those SMRs expressed in % as $EM_{j}^{y,z}=(100∙m_{j}^{y,s}/m_{j}^{z,s}-100)$. A 95%-confidence interval (95%-CI) was obtained via the delta method as [25]:

$$95\%CI(EM_{j}^{y,z})=100exp\left\{log(\frac{m_{j}^{y,s}}{m_{j}^{z,s}})\pm 1.96\sqrt{\sum_{t=y,z}\frac{\sum_{i=0}^{100}D_{ij}^{t}{\left(P_{ij}^{s}/P_{ij}^{t}\right)}^{2}}{{\left(\sum_{i=0}^{100}D_{ij}^{t}P_{ij}^{s}/P_{ij}^{t}\right)}^{2}}}\right\}-100.$$

Similarly, an EM between an observed and a predicted SMR for a given year *y* was calculated for each sex *j* by: $EM_{j}^{y,\hat{y}}=(100∙m_{j}^{y,s}/{\hat{m}}_{j}^{y,s}-100)$, with 95%-confidence interval:

$$95\%CI(EM_{j}^{y,\hat{y}})=100∙exp\left\{log(\frac{m_{j}^{y,s}}{{\hat{m}}_{j}^{y,s}})\pm 1.96\sqrt{\frac{\sum_{i=0}^{100}D_{ij}^{y}{\left(P_{ij}^{s}/P_{ij}^{y}\right)}^{2}}{{\left(\sum_{i=0}^{100}D_{ij}^{y}P_{ij}^{s}/P_{ij}^{y}\right)}^{2}}+V[\log\;{\hat{m}}_{j}^{y,s}]}\right\}-100.$$

Similar calculations were done for EMs in specific age classes.

### Excess deaths

An Excess Deaths (ED) between one given year *y* and a past year *z* was calculated for each sex *j* by subtracting the number of deaths of those two years, standardized to the reference population: $ED_{j}^{y,z}={(m}_{j}^{y,s}P_{j}^{s}-m_{j}^{z,s}P_{j}^{s})$. Similarly, an ED between an observed and a predicted number of deaths for a given year *y* was defined for each sex *j* as: $ED_{j}^{y,\hat{y}}=(m_{j}^{y,s}P_{j}^{s}-{\hat{m}}_{j}^{y,s}P_{j}^{s})$. Similar calculations were done for EDs in specific age classes.

### Life expectancy at birth and life expectancy losses

Life Expectancy at birth (LE) was obtained via a piecewise exponential model [26] using numbers of deaths (and population sizes) stratified in 19 age classes (5-year age classes, with a last open class of 90+), i.e. using for each year the age classes available in 2022. Let *I*_*k*_ (*k* = 1,…,19) be the age classes: 0–4, 5–9, … 80–84, 85–89, 90+, and $\mu_{I_{k}j}^{y}$ the (crude) mortality rate in age class *I*_*k*_: $\mu_{I_{k}j}^{y}=\sum_{i\in I_{k}}D_{ij}^{y}$/$\sum_{i\in I_{k}}P_{ij}^{y}$. Let then ${\dot{m}}_{ij}^{y}=\mu_{I_{k}j}^{y}$, where *k* is taken such that *i*∈*I*_*k*_, define the mortality rate for age *i* (*i* = 0,…,100), assumed constant within each age class. The LE of year *y* can be calculated for each sex *j* as follows (derivation shown in Supplementary material of [5]):

$$e_{j}^{y}=\frac{1}{2}+\sum_{l=0}^{110}exp(-\sum_{i=0}^{l}{\dot{m}}_{ij}^{y}).$$

A Life Expectancy Loss (LEL) between one given year *y* and a past year *z* was then calculated in months as ${LEL}_{j}^{y,z}=12∙{(e}_{j}^{y}{-e}_{j}^{z})$. A predicted LE for year *y* (based on the above estimated trend) and sex *j*, ${\hat{e}}_{j}^{y}$, was calculated by replacing in these formulae the observed $\mu_{I_{k}j}^{y}$ with the predicted ones, obtained from the predicted sex- and age-specific mortality rates: ${\hat{\mu }}_{I_{k}j}^{y}=\sum_{i\in I_{k}}{\hat{m}}_{ij}^{y}P_{ij}^{y}/\sum_{i\in I_{k}}P_{ij}^{y}$. A LEL comparing an observed and a predicted life expectancy for a given year *y* was then obtained for each sex *j* as: ${LEL}_{j}^{y,\hat{y}}=12∙{(e}_{j}^{y}-{\hat{e}}_{j}^{y})$. Similar calculations were made to obtain the (residual) life expectancy and the (residual) life expectancy loss at age 50.

All calculation were made in the software R-4.3.0 [27].

### Results

**Fig 1:**
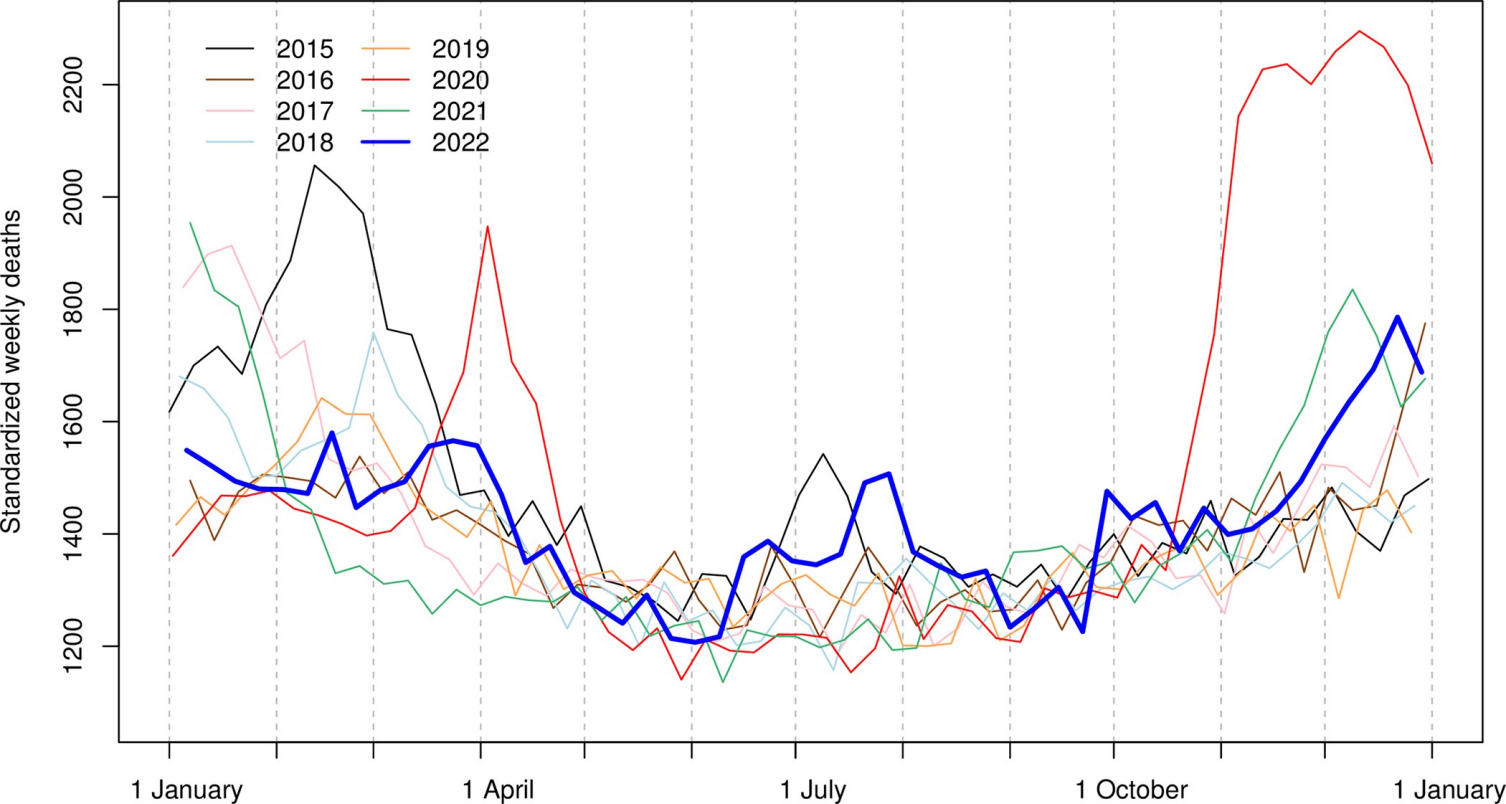
Standardized weekly deaths in Switzerland for the period 2015–22. Reference for standardization: January 1^st^, 2022 (data from the Swiss Federal Statistical Office—FSO).

Fig 1 shows the standardized weekly deaths (SWD) for the period 2015–22. After a first pandemic year (2020) characterized by two large waves of deaths, particularly the second in autumn, and a second year (2021) marked by a first half of low SWDs and a wave at the end of the year, SWDs in the third pandemic year (2022) were overall within the range of pre-pandemic years. In particular, no wave of deaths was seen during the typical winter period for seasonal influenza, whereas only a modest wave was seen between late winter and early spring. On the other hand, an unusual wave of deaths was observed during the summer, similar in magnitude to that observed in 2015, with a first peak in June and a second peak in late July, while another increase of SWDs was observed at the end of the year. S1 Fig shows how the 2022 mortality waves relate to COVID-19 incidence and COVID-19 attributed deaths for that year in Switzerland (data from the Federal Office of Public Health COVID-⁠19 Suisse | Coronavirus | Dashboard (admin.ch)). A curve of peak temperatures from May to September measured among three Swiss meteorological stations (Zurich, Geneva and Basel) is also added to the graph (data from Climatologie par jour des stations météo (prevision-meteo.ch)). Summing up the weekly deaths, with a correction to match the period from January 1 to December 31, 2022 (Data and methods Section), we had a total 74’300 deaths for 2022 (at the last access on June 13, 2023), compared to 71’192 in 2021, 76’195 in 2020 and 67’780 in 2019. The population sizes were of 8’544’527, 8’606’033, 8’670’300 and 8’738’791 between 2019 and 2022, corresponding to an increase of 0.7–0.8% each year.

**Fig 2:**
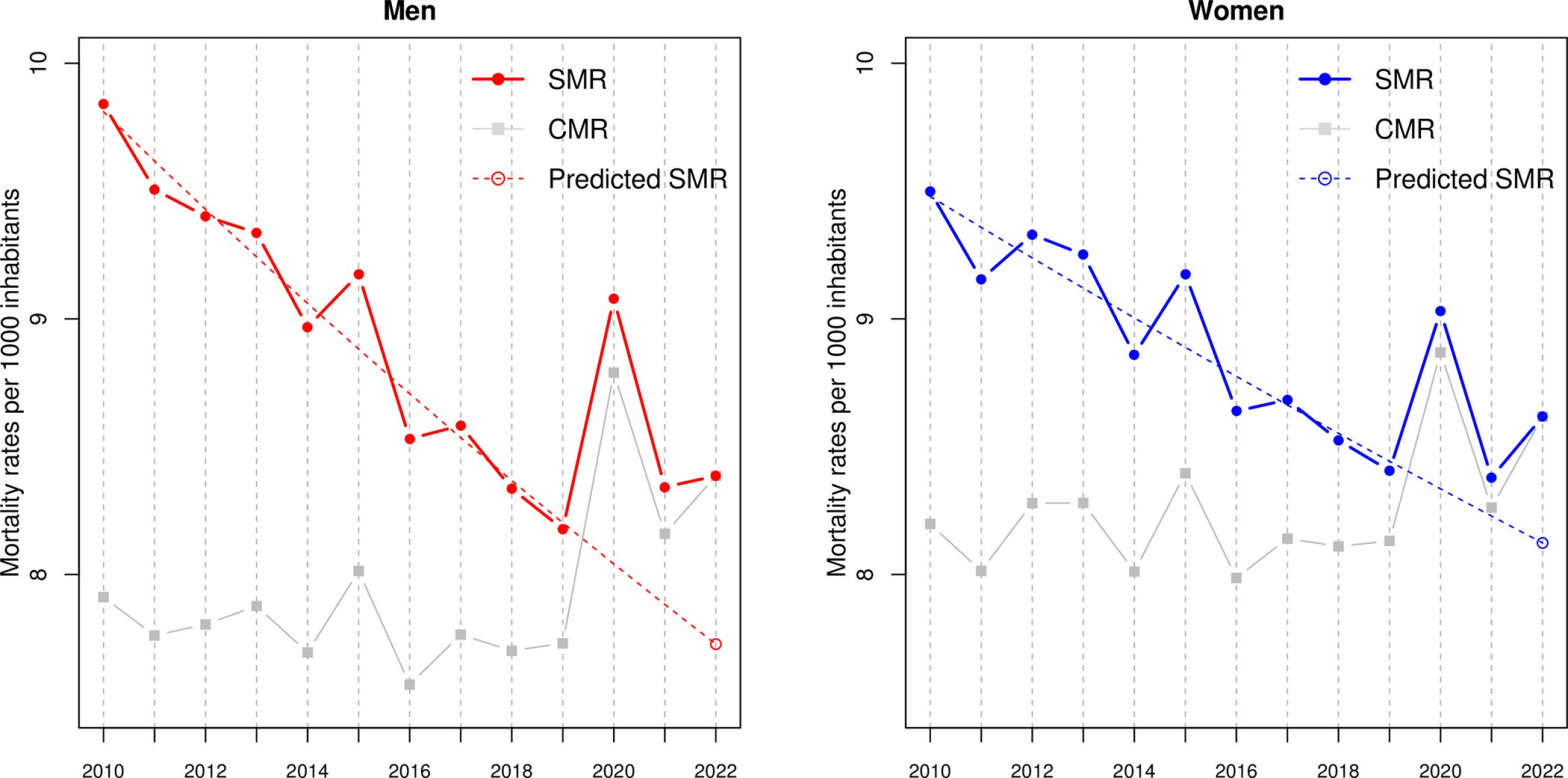
Crude mortality rates (CMR) and standardized mortality rates (SMR) by sex in Switzerland for the period 2010–22, together with predicted SMR taking into account the pre-pandemic trend estimated over the period 2010–19. Reference for standardization: January 1^st^, 2022 (data from the Swiss Federal Statistical Office—FSO).

Crude mortality rates (CMR) and standardized mortality rates (SMR) are plotted in Fig 2 for the period 2010–22 separately for both sexes, along with the predicted SMR taking into account the downward trend estimated over a 10-year pre-pandemic period (Data and method Section). While CMRs remained roughly stable during the pre-pandemic period, SMRs were steadily declining, especially for men, leading to a low predicted SMR in 2022. Observed and estimated trends for SMRs of both sexes by 10-year age classes (last open class of 90+) provided by our model can be seen in S2 Fig. Observed SMRs were declining in every age class, with more pronounced fluctuations for the younger age classes due to the small numbers of deaths. One can check that our model fitted pre-pandemic SMRs very well in each age class. On the other hand, for both sexes (and most age classes), the observed SMRs were clearly above the predicted ones during the pandemic years, indicating that the trend was lost from 2020 onward.

Table 1 provides excess mortality (EM) and excess deaths (ED) when comparing the SMR of a given pandemic year (2020–2022) with that of the last pre-pandemic year 2019. After having increased in 2020 by 11.0% (95%-CI: 9.4; 12.7) for men and 7.4% (95%-CI: 5.9; 9.0) for women compared to 2019, SMRs largely (but not entirely) recovered the pre-pandemic level in 2021–22 for both sexes, with still an EM of 2.6% (95%-CI: 1.0; 4.1) for men and 2.5% (95%-CI: 1.0; 4.0) for women in 2022 compared to 2019. This corresponds to an excess of 905 deaths for men and 930 deaths for women.

**Table 1:** Excess mortality (EM in %), together with 95%-CI, excess deaths (ED in numbers), and life expectancy losses (LEL and LEL_50_ in months) by sex in Switzerland when comparing standardized mortality rate (SMR), respectively life expectancies (LE and LE_50_), a given year (2020, 2021 or 2022) with that of 2019, or when comparing observed and predicted SMR (respectively LE and LE_50_) in 2022, taking into account the pre-pandemic trend estimated over the period 2010–19. Reference for standardization: January 1^st^, 2022 (data from the Swiss Federal Statistical Office—FSO).

	**MEN**				
	**EM (%)**	**95%CI (%)**	**ED (n)**	**LEL (m)**	**LEL**_**50**_ **(m)**
2020 vs 2019	11.0	(9.4 ; 12.7)	3913	-10.1	-9.0
2021 vs 2019	2.0	(0.5; 3.6)	715	-3.2	-2.7
2022 vs 2019	2.6	(1.0; 4.1)	905	-2.7	-1.6
2022 vs trend	8.4	(6.9; 9.9)	2826	-8.8	-7.5
	**WOMEN**				
	**EM (%)**	**95%CI (%)**	**ED (n)**	**LEL (m)**	**LEL**_**50**_ **(m)**
2020 vs 2019	7.4	(5.9 ; 9.0)	2745	-5.8	-5.2
2021 vs 2019	-0.3	(-1.8 ; 1.2)	-118	0.2	0.1
2022 vs 2019	2.5	(1.0; 4.0)	930	-2.4	-1.7
2022 vs trend	6.0	(4.6; 7.5)	2160	-6.0	-4.9

Table 1 also provides estimated EMs and EDs obtained when comparing SMRs in 2022 to those predicted by our model, had the pre-pandemic declining trend continued during the pandemic years. We found an EM of 8.4% (95%-CI: 6.9; 9.9) for men and of 6.0% (95%-CI: 4.6; 7.5) for women, the former being larger than the latter due to the steeper pre-pandemic trend estimated for men than for women. This corresponds to an excess of 2826 deaths for men and 2160 deaths for women, respectively 3.1 and 2.3 more than when comparing the SMRs of 2022 and 2019.

**Fig 3:**
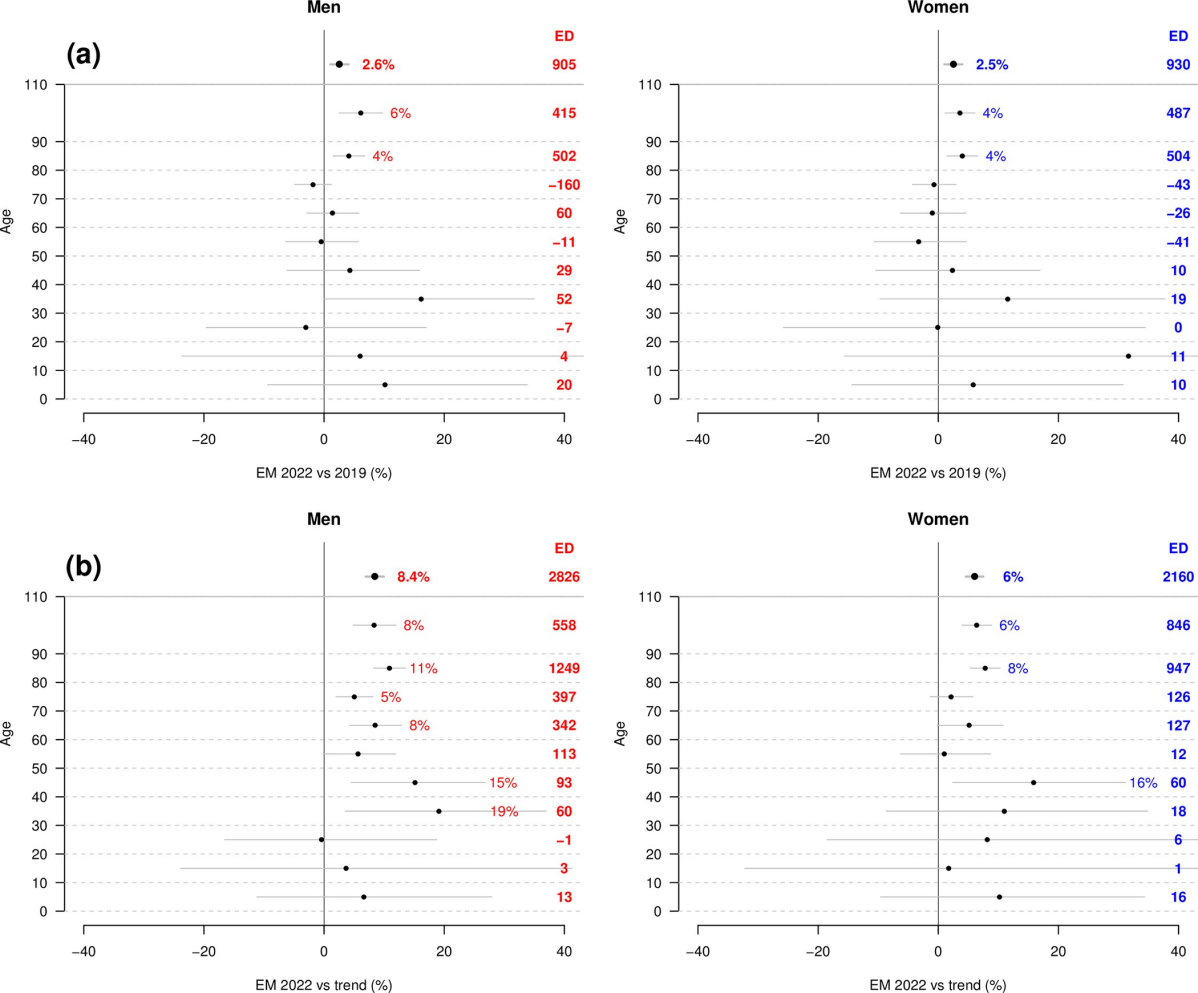
Excess mortality (EM) and excess deaths (ED) by sex and 10-year age classes in Switzerland, (a) when comparing standardized mortality rate (SMR) in 2022 and 2019, (b) when comparing observed and predicted SMR in 2022, taking into account the pre-pandemic trend estimated over the period 2010–19. EMs are printed when they reach statistical significance at the 5% significance level, i.e. the 95%CI do not cover the zero line. Reference for standardization: January 1^st^, 2022 (data from the Swiss Federal Statistical Office—FSO).

Fig 3 shows the EMs (with 95% CI) and EDs stratified by sex and 10-year age classes (last open class of 90+), where the SMRs of 2022 are compared either to the pre-pandemic level of 2019 (Panels a), or to that predicted by the pre-pandemic trend (Panels b). Compared to the pre-pandemic level of 2019, significant EMs were found over age 80, corresponding to an excess of 917 deaths for men and 991 deaths for women in these age classes. An almost significant EM was also observed for men in the 30–40 age class, and a rising tendency was observed in the same age class for women, also this contributed to an excess of “only” 52 and 19 deaths, respectively. On the other hand, compared to the pre-pandemic trend, EMs were significant for men in most age classes over 30. In women, significance was reached in the age classes of 40–50 and 80+. The strong and significant excess mortality compared with the estimated trend for both sexes at ages 40–50 is related to the steep downward trend estimated by our model for this age class (steeper than in other age classes, S2 Fig).

**Fig 4:**
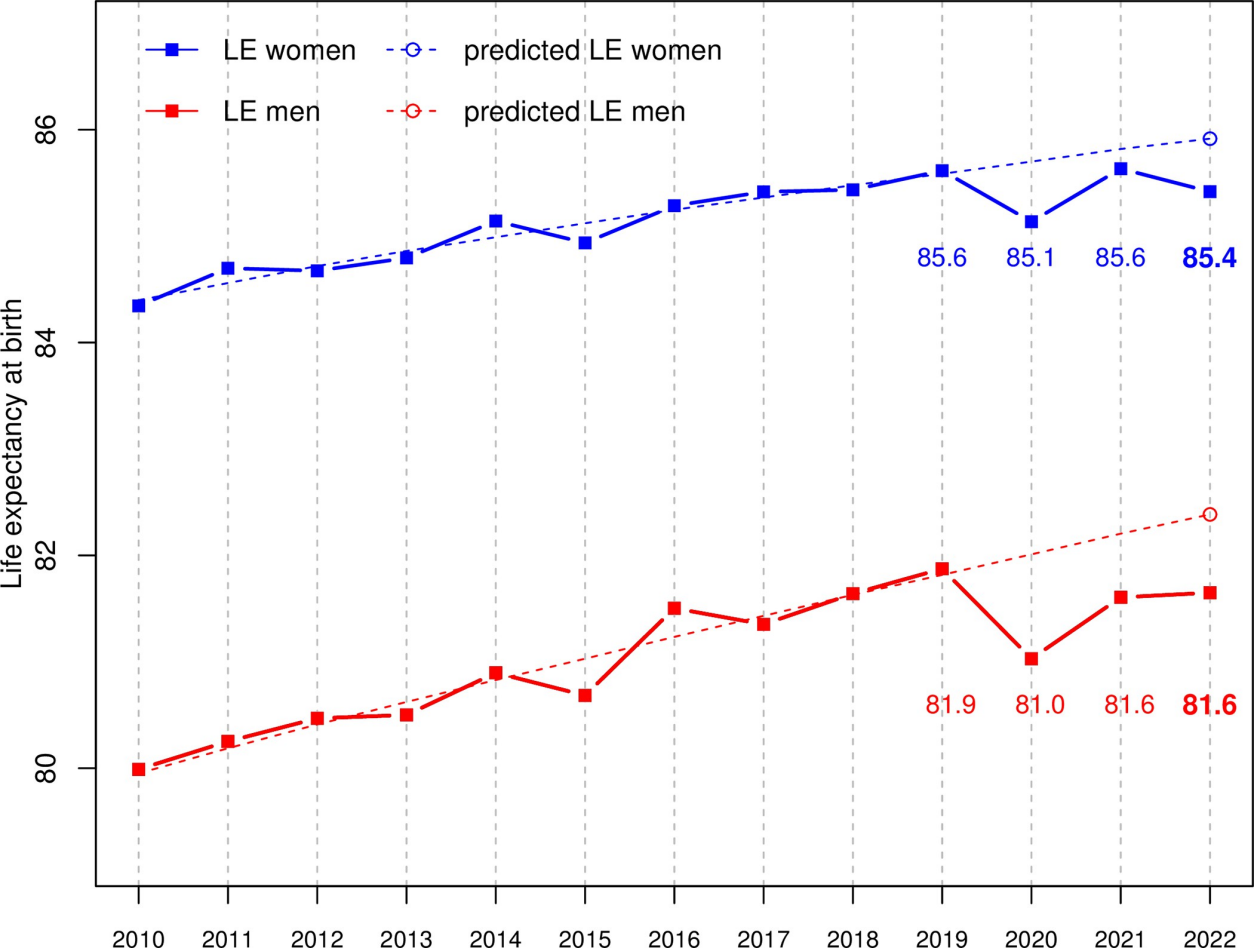
Life expectancy (LE) by sex in Switzerland for the period 2010–22, together with their predicted values taking into account the pre-pandemic trend estimated over the period 2010–19 (data from the Swiss Federal Statistical Office—FSO).

Life expectancy at birth (LE) for the period 2010–22 is presented in Fig 4, along with the predicted LE resulting from our model (Data and methods Section). After having decreased in 2020 by 10.1 months for men and 5.8 months for women compared to 2019, LE largely (but not entirely) recovered the pre-pandemic level in 2021–22 for both sexes, with LEL still of 2.7 months for men and 2.4 months for women in 2022 compared to 2019 (Fig 4 and Table 1). A similar pattern was found for the residual life expectancy at age of 50 (Table 1). On the other hand, LEL was considerably larger, -0.73 years (-8.8 months) for men and -0.5 years (-6.0 months) for women, when comparing LE in 2022 with the predicted one (Fig 4 and Table 1). LELs in 2022 thus appear to be 3.3 (for men) and 2.5 (for women) higher with respect to past trends than with respect to past levels, much like what was observed for EDs in 2022.

## Discussion

Three years after the onset of the COVID-19 pandemic, and although based on mortality data that are not yet fully definitive, our analyses have shown that mortality in Switzerland in 2022 was still slightly above that of the last pre-pandemic year, with an increase of the standardized mortality rate of 2.5% compared to 2019, corresponding to global excess of 1835 deaths, mainly due to the age classes over 80, and a loss of life expectancy of about 2.5 months, with similar results for men and women.

The reasons for this excess mortality of the elderly is an open question. COVID-19 remains at stake, with 1882 deaths officially attributed to COVID-19 in Switzerland in 2022 according to the Federal Office of Public Health (COVID-⁠19 Suisse | Coronavirus | Dashboard (admin.ch)), mainly among the elderly. This is however much less than the 7610 and 4335 deaths attributed to COVID-19 in 2020 and 2021. Moreover, the weekly number of deaths observed throughout 2022 does not appear to be correlated with the COVID-19 attributed deaths that year (S1 Fig), unlike in 2020 and (to a lesser extent) in 2021 [5]. Another potential cause is the heavy heat wave that hit Europe and Switzerland in the summer of 2022 (Surface air temperature for August 2022 | Copernicus), which temporally corresponded to the waves of deaths observed in the summer of that year (S1 Fig). In Switzerland, it has been estimated that the heat waves of the summers of 2003 and 2015 were responsible for an excess of respectively 975 and 804 deaths, mainly among people aged 75 or more [28]. The return of influenza at the end of 2022 (Grippe saisonnière: rapport de situation en Suisse (admin.ch)), after an absence of almost two years, could be behind the wave of deaths observed at the end of the year. A combination of the three causes is also possible.

Whether based on excess mortality or life expectancy loss, the situation appears more dramatic if one compares the observed mortality in 2022 with the mortality expected had the declining pre-pandemic trend continued during the three years of the pandemic, i.e. using the second approach mentioned in the Introduction. While we estimated an excess of 1835 deaths (905 men and 930 women) in Switzerland in 2022 compared to 2019 (+2.6% for men and +2.5% for women), our estimate rose to 4986 deaths (2826 for men and 2160 women) when taking into account the trend (+8.4% for men and +6.0% for women), which is almost three times more. Similarly, while the loss of life expectancy in 2022 was only 2.7 months for men and 2.4 months for women compared to 2019, it reached 8.8 months for men and 6.0 months for women when taking into account the trend. The higher excess mortality (and greater life expectancy loss) for men than for women when using the second approach is because the estimated pre-pandemic trend was more pronounced for men than for women, the former catching up the latter. Likewise, while EMs were significant only in age classes 80+ using the first approach, they were also significant in most age classes over 30 for men, and in age class 40–50 for women, using the second approach. These results are consistent with those presented for France in a recent report by the INSEE (National Institute of Statistics and Economic Studies) [29], highlighting a higher mortality in 2022 than that expected according to the 2010–19 trend in all age classes, particularly for the under-55s and over-85s.

The two approaches could also be applied to other metrics than standardized mortality rates or life expectancy to estimate the impact of a pandemic on overall mortality. For example, some authors underlined that a life expectancy loss is likely to exaggerate the impact of a pandemic, since life expectancy in a pandemic year is defined as the average life span of a hypothetical cohort of people who would live their entire lives under the mortality conditions observed that year, i.e. with the pandemic [30]. A concept of “population life loss” has thus been proposed to measure the impact of a pandemic on real (rather than hypothetical) populations [31]. But whatever the metrics that is used, we would obtain more dramatic results with the second approach than with the first.

Since the results are clearly not the same using the two approaches, the question arises whether it makes more sense to compare mortality of a given year with past mortality levels or with past mortality trends. This question is directly related to what we expect from the future of human longevity, the first approach certainly being relevant if we do not believe in indefinite progress in this regard, the second being sensible if we do. The situation is not necessarily the same in all countries. While life expectancy at birth tended to increase before the pandemic in most industrialized countries, including Switzerland [32–34], some stagnation has been observed in recent years in other countries such as the United States, Denmark, the Netherlands and the United Kingdom [35–39]. Even in the former countries, the increasing trend in life expectancy may not last forever. Mortality levels were already so low and life expectancy so high in Switzerland and other industrialized countries before the pandemic that we may be approaching some limits of human progress in terms of survival [40, 41], especially if phenomena other than COVID-19 begin or continue to impact mortality in the coming years, such as summer heat waves, as pointed out by the INSEE [29]. Other authors are more optimistic, such as demographer James Vaupel, an advocate of continued progress in human survival into older ages, who stated in 2021: “With the novel COVID-19 illness, new scenarios may arise, but it is still uncertain how the pandemic will affect the longevity in the future: although it may have a short term impact on life expectancy similar to the Spanish flu in 1918, its effects could be small or even positive in the longer term thanks to behavioral and policy changes” [42]. Paradoxically, these kinds of optimistic statements about the future of human longevity will support a mortality comparison to past trends rather than past levels, and ultimately lead to more pessimistic statements regarding excess mortality in a given year.

Although it occurred more than 100 years ago, at a time when life expectancy in Switzerland was around 55 years [Human Mortality Database], and although it was much more deadly than COVID-19, the example of the Spanish Flu remains of interest. Life expectancy decreased by more than 10 years for Swiss men and more than 8 years for Swiss women in 1918 compared to 1917, which represents a relative loss of about 20 times that observed in 2020, the first year of the COVID-19 pandemic, compared to 2019 [34]. Yet life expectancy increased back the year after (in 1919) by respectively 10 and 7 years, and after another year of relative stagnation (1920), it regained its upward trend already in 1921, with a life expectancy exceeding that of 1917 by two years for both sexes [Human Mortality Database]. Whether we will see a similar pattern for the pandemic of COVID-19 is a current issue. So far, our analysis suggested that the pre-pandemic trend has been lost in 2022 not only for the older age classes, which were most impacted by COVID-19 during the pandemic years, but also for the younger age classes, which were not. It will thus be interesting to see whether the upward trend in life expectancy in Switzerland will be regained in 2023 or later years, retrospectively justifying the use of the second approach to estimate excess mortality in years 2020–22, or whether it might be lost, favoring the first approach.

One difficulty with the second approach is that many models can be (and have been) used to estimate a pre-pandemic trend, based on different assumptions and using different time periods, with potentially different results. Whatever the model used, there might always be criticism and doubts. For example, a recent study [43] compared several models on simulated data, highlighting the risks associated with the use of complex models, particularly spline models with too many parameters. This study also warns against the choice of time period for estimation, indicating, for example, that starting the estimation period in 2015, a year of excess mortality in many countries, can lead to poor results. In robust statistics, an outlier at one extreme of the observation period is known to be potentially capable of distorting results, acting as a bad leverage point [44]. These two reasons appear to be behind the WHO’s overestimation of excess mortality, as demonstrated for Germany by what was called the “German puzzle” [45]. On the Swiss data, we were able to verify that, by starting the estimation period in 2015, a sharper trend would be estimated compared to all the other starting years between 2005 and 2014, including our choice of 2010, all of which providing an excellent fit with approximately the same estimated trend. We may also note that in the context of an international comparison, the second approach would require a possibly different model in each country, which could complicate communication, interpretation, and even the credibility of the results. This is perhaps one of the reasons why the studies [21, 22] that calculated the life expectancy loss in 35 countries have indeed used the first approach, comparing life expectancy in 2020 and 2021 with that of 2019.

Another issue when modeling mortality is the time granularity, i.e. whether annual, monthly or weekly data should be used for estimating the trend. Indeed, most studies cited in the Introduction used weekly or monthly mortality data, including a seasonal effect (e.g. via sine-cosine functions) in their models [11–16]. This was not possible in the present study since we did not have weekly or monthly data with sufficient sex and age detail. This could be seen as a limitation of our work. However, in Poisson (as in linear) regression, adjusting for covariates that are not correlated with an exposure of interest will not affect the estimate of the exposure effect [46, 47], so that we would obtain the same expected annual mortality with or without adjustment for a seasonal effect in our model. Since, in the latter case, one could equivalently aggregate weekly or monthly mortality data into annual mortality data (another property of Poisson regression), we would in fact obtain the same annual results using either weekly, monthly or annual data granularity.

As considered in [43], the first approach could be seen as a special case of the second, in which the pre-pandemic trend is assumed to stop at the onset of the pandemic, an assumption that is certainly unrealistic after years of clear downward trend. However, the first approach can also be seen as a mere comparison with the past, which is a natural reference, without any claim to predict the future. In other words, the first approach is a *factual comparison with what has happened*, while the second is a *counterfactual comparison with what could have happened* without a pandemic, corresponding to two different philosophical point of views. The second approach is thus inevitably speculative, whereas the first needs not be.

To conclude, our purpose is not to make a definitive judgment on which approach is generally more suitable to estimate an excess of mortality, but to raise awareness of their differences in philosophy and results. In fact, both approaches can also be used simultaneously, providing two complementary pieces of information. For Switzerland, the second approach enabled us to show that the downward pre-pandemic trend in mortality is currently halted, a clearly important result. However, this statement alone may mask another important message, namely that the low pre-pandemic mortality levels and the high pre-pandemic life expectancy have largely been recovered by 2022, as highlighted by the first approach.

## Supporting information

S1 Fig Standardized weekly deaths in Switzerland for the period 2015–22. (Reference for standardization: January 1^st^, 2022, data from the Swiss Federal Statistical office—FSO), together with COVID-19 incidence and COVID-19 attributed deaths for the year 2022 (data from the Federal Office of Public Health—OFSP) and a temperature indicator curve giving peak temperatures among three major Swiss weather stations (data from PrevisionMeteo.ch).
(TIF)

S2 Fig Standardized mortality rates (SMR) by sex and 10-year age classes (last open class 90+) in Switzerland for the period 2010–22, together with their predicted values taking into account the pre-pandemic trends estimated over the period 2010–19. Reference for standardization: January 1^st^, 2022 (data from the Swiss Federal Statistical Office—FSO).
(TIF)

## References

[1] Beaney T, Clarke JM, Jain V, Golestaneh AK, Lyons G, Salman D, et al. Excess mortality: the gold standard in measuring the impact of COVID-19 worldwide? *J R Soc Med*. 2020 Sep;113(9):329–334. doi: 10.1177/0141076820956802 32910871 PMC7488823

[2] COVID-19 Excess Mortality Collaborators. Estimating excess mortality due to the COVID-19 pandemic: a systematic analysis of COVID-19-related mortality, 2020–21. *Lancet*. 2022 Apr 16;399(10334):1513–1536. doi: 10.1016/S0140-6736(21)02796-3 35279232 PMC8912932

[3] Msemburi W, Karlinsky A, Knutson V, Aleshin-Guendel S, Chatterji S, Wakefield J. The WHO estimates of excess mortality associated with the COVID-19 pandemic. *Nature*. 2023 Jan;613(7942):130–137. doi: 10.1038/s41586-022-05522-2 36517599 PMC9812776

[4] Locatelli I, Rousson V. A first analysis of excess mortality in Switzerland in 2020. *PLoS One*. 2021 Jun 17;16(6):e0253505. doi: 10.1371/journal.pone.0253505 34138948 PMC8211252

[5] Locatelli I, Rousson V. Mortality in Switzerland in 2021. *PLoS One*. 2022 Sep 9;17(9):e0274295. doi: 10.1371/journal.pone.0274295 36084010 PMC9462753

[6] Ahmad FB, Cisewski JA, Miniño A, Anderson RN. Provisional Mortality Data—United States, 2020. *MMWR Morb Mortal Wkly Rep*. 2021 Apr 9;70(14):519–522. doi: 10.15585/mmwr.mm7014e1 33830988 PMC8030985

[7] Joy M, Hobbs FR, Bernal JL, et al. Excess mortality in the first COVID pandemic peak: cross-sectional analyses of the impact of age, sex, ethnicity, household size, and long-term conditions in people of known SARS-CoV-2 status in England. *Br J Gen Pract*. 2020;70(701):e890–e898. Published 2020 Nov 26. doi: 10.3399/bjgp20X713393 33077508 PMC7575407

[8] Alicandro G, Remuzzi G, La Vecchia C. Italy’s first wave of the COVID-19 pandemic has ended: no excess mortality in May, 2020. *Lancet*. 2020;396(10253):e27–e28. doi: 10.1016/S0140-6736(20)31865-1 32891216 PMC7470816

[9] Kelly G, Petti S, Noah N. Covid-19, non-Covid-19 and excess mortality rates not comparable across countries. *Epidemiol Infect*. 2021;149:e176. Published 2021 Aug 2. doi: 10.1017/S0950268821001850 34338184 PMC8365039

[10] Vieira A, Peixoto VR, Aguiar P, Abrantes A. Rapid Estimation of Excess Mortality during the COVID-19 Pandemic in Portugal -Beyond Reported Deaths. *J Epidemiol Glob Health*. 2020;10(3):209–213. doi: 10.2991/jegh.k.200628.001 32954711 PMC7509098

[11] Ghafari M, Kadivar A, Katzourakis A. Excess deaths associated with the Iranian COVID-19 epidemic: A province-level analysis. *Int J Infect Dis*. 2021;107:101–115. doi: 10.1016/j.ijid.2021.04.015 33862214 PMC8208896

[12] Kobak D. Excess mortality reveals Covid’s true toll in Russia. *Signif (Oxf)*. 2021;18(1):16–19. doi: 10.1111/1740-9713.01486 33821160 PMC8013319

[13] Aburto JM, Kashyap R, Schöley J, et al. Estimating the burden of the COVID-19 pandemic on mortality, life expectancy and lifespan inequality in England and Wales: a population-level analysis. *J Epidemiol Community Health*. 2021;75(8):735–740. doi: 10.1136/jech-2020-215505 33468602 PMC7818788

[14] Islam N, Shkolnikov VM, Acosta RJ, et al. Excess deaths associated with covid-19 pandemic in 2020: age and sex disaggregated time series analysis in 29 high income countries. *BMJ*. 2021;373:n1137. Published 2021 May 19. doi: 10.1136/bmj.n1137 34011491 PMC8132017

[15] Woolf SH, Chapman DA, Sabo RT, Zimmerman EB. Excess Deaths From COVID-19 and Other Causes in the US, March 1, 2020, to January 2, 2021. *JAMA*. 2021 Apr 2;325(17):1786–9. doi: 10.1001/jama.2021.5199 33797550 PMC8019132

[16] Staub K, Panczak R, Matthes KL, Floris J, Berlin C, Junker C, et al. Historically High Excess Mortality During the COVID-19 Pandemic in Switzerland, Sweden, and Spain. *Ann Intern Med*. 2022 Apr;175(4):523–532. doi: 10.7326/M21-3824 35099995 PMC8803137

[17] Luy M, Di Giulio P, Di Lego V, Lazarevič P, Sauerberg M. Life Expectancy: Frequently Used, but Hardly Understood. *Gerontology*. 2020; 66(1):95–104. doi: 10.1159/000500955 31390630 PMC7026938

[18] Lee R. D., Carter L. R., Modeling and forecasting US mortality. J. Am. Stat. Assoc. 1992; 87, 659–671. doi: 10.2307/2290201

[19] Li N, Lee R. Coherent mortality forecasts for a group of populations: an extension of the Lee-Carter method. *Demography*. 2005 Aug;42(3):575–94. doi: 10.1353/dem.2005.0021 16235614 PMC1356525

[20] Booth H., Hyndman R. J., Tickle L., De Jong P., Lee-Carter mortality forecasting: A multi-country comparison of variants and extensions. *Demogr*. *Res*. 2006; 15, 289–310. doi: 10.4054/DemRes.2006.15.9

[21] Aburto JM, Schöley J, Kashnitsky I, Zhang L, Rahal C, Missov TI, et al. Quantifying impacts of the COVID-19 pandemic through life-expectancy losses: a population-level study of 29 countries. *Int J Epidemiol*. 2022 Feb 18;51(1):63–74. doi: 10.1093/ije/dyab207 34564730 PMC8500096

[22] Schöley J, Aburto JM, Kashnitsky I, Kniffka MS, Zhang L, Jaadla H, et al. Life expectancy changes since COVID-19. *Nat Hum Behav*. 2022 Dec;6(12):1649–1659. doi: 10.1038/s41562-022-01450-3 36253520 PMC9755047

[23] Fleiss JL, Levin B, Cho Paik M. Statistical methods for rates and proportions 2003. Wiley Series in Probability and Statistics Eds. doi: 10.1002/0471445428

[24] Cameron AC, & Trivedi PK. Regression Analysis of Count Data, 2013 (Second Edition). Cambridge University Press. 10.1017/CBO9781139013567.

[25] Fay MP. Approximate confidence intervals for rate ratios from directly standardized rates with sparse data. *Communications in Statistics—Theory and Methods* 1999, 28: 2141–2160. doi: 10.1080/03610929908832411

[26] Friedman M (1982). Piecewise exponential models for survival data with covariates. *Annals of Statistics*, 10, 101–113. doi: 10.1214/aos/1176345693

[27] R Core Team (2022). R: A language and environment for statistical computing. R Foundation for Statistical Computing, Vienna, Austria. URL https://www.R-project.org/.

[28] Vicedo-Cabrera AM, Ragettli MS, Schindler C, Röösli M. Excess mortality during the warm summer of 2015 in Switzerland. Swiss Med Wkly. 2016 Dec 5; 146:w14379. doi: 10.4414/smw.2016.14379 31309989

[29] Insee (2023). 53 800 décès de plus qu’attendus en 2022: une surmortalité plus élevée qu’en 2020 et 2021. *Insee Première*, n° 1951, Juin 202.

[30] Goldstein JR, Lee RD. Demographic perspectives on the mortality of COVID-19 and other epidemics. *Proc Natl Acad Sci* U S A. 2020 Sep 8;117(36):22035–22041. doi: 10.1073/pnas.2006392117 Epub 2020 Aug 20. 32820077 PMC7486771

[31] Rousson V, Locatelli I. On the impacts of the COVID-19 pandemic on mortality: Lost years or lost days? *Front Public Health*. 2022 Nov 9;10:1015501. doi: 10.3389/fpubh.2022.1015501 36438204 PMC9682259

[32] Janssen F, Bardoutsos A, El Gewily S, De Beer J. Future life expectancy in Europe taking into account the impact of smoking, obesity, and alcohol. *Elife*. 2021 Jul 6;10:e66590. doi: 10.7554/eLife.66590 34227469 PMC8337079

[33] Kontis V, Bennett JE, Mathers CD, Li G, Foreman K, Ezzati M. Future life expectancy in 35 industrialised countries: projections with a Bayesian model ensemble. *Lancet*. 2017 Apr 1;389(10076):1323–1335. doi: 10.1016/S0140-6736(16)32381-9 28236464 PMC5387671

[34] Rousson V, Paccaud F, Locatelli I. Comparing the loss of life expectancy at birth during the 2020 and 1918 pandemics in six European countries. *Vienna Yearbook of Population Research*, 2022. 1: 527–542. doi: 10.1553/populationyearbook2022.dat.7

[35] Denney JT, McNown R, Rogers RG, Doubilet S. Stagnating Life Expectancies and Future Prospects in an Age of Uncertainty. *Soc Sci Q*. 2013 Jun;94(2):445–461. doi: 10.1111/j.1540-6237.2012.00930.x 25506092 PMC4264628

[36] Harper S, Riddell CA, King NB. Declining Life Expectancy in the United States: Missing the Trees for the Forest. *Annu Rev Public Health*. 2021 Apr 1;42:381–403. doi: 10.1146/annurev-publhealth-082619-104231 33326297

[37] Kallestrup-Lamb M, Kjaergaard S, Rosenskjold CPT. Insight into stagnating adult life expectancy: Analyzing cause of death patterns across socioeconomic groups. *Health Econ*. 2020 Dec;29(12):1728–1743. doi: 10.1002/hec.4166 32969122

[38] Walsh D, McCartney G, Minton J, Parkinson J, Shipton D, Whyte B. Changing mortality trends in countries and cities of the UK: a population-based trend analysis. *BMJ Open*. 2020 Nov 5;10(11):e038135. doi: 10.1136/bmjopen-2020-038135 33154048 PMC7646340

[39] Kaneda T, Scommegna P. Trends in Life Expectancy in the United States, Denmark, and the Netherlands: Rapid Increase, Stagnation, and Resumption. *Population Reference Bureau*. Today’s Research on Aging 2011, No. 22.

[40] Fries JF. Aging, natural death, and the compression of morbidity. *N Engl J Med*. 1980 Jul 17;303(3):130–5. doi: 10.1056/NEJM198007173030304 7383070

[41] Dong X, Milholland B, Vijg J. Evidence for a limit to human lifespan. *Nature*. 2016 Oct 13;538(7624):257–259. doi: 10.1038/nature19793 27706136 PMC11673931

[42] Vaupel JW, Villavicencio F, Bergeron-Boucher MP. Demographic perspectives on the rise of longevity. *Proc Natl Acad Sci* USA. 2021 Mar 2;118(9):e2019536118. doi: 10.1073/pnas.2019536118 33571137 PMC7936303

[43] Ferenci T. Comparing methods to predict baseline mortality for excess mortality calculations–unravelling ‘the German puzzle’ and its implications for spline-regression. *MedRxiv* 10.1101/2022.07.18.22277746. PMC10585880 37853374

[44] Rousseew PJ and Van Zomeren BC. Unmasking Multivariate Outliers and Leverage Points. *Journal of the American Statistical Association* 1990. 85(411):633–639 doi: 10.1080/01621459.1990.10474920

[45] Van Noorden R. COVID death tolls: scientists acknowledge errors in WHO estimates. *Nature*. 2022;606(7913):242–244. doi: 10.1038/d41586-022-01526-0 35650295

[46] Gail MH, Wieand S, Piantadosi S. Biased estimates of treatment effect in randomized experiments with nonlinear regression s and omitted covariates. *Biometrika* 1984, 71(3), 431–33.

[47] Robinson LD, Dorroh JR, Lien D, Tiku ML. The effects of covariate adjustment in generalized linear models. *Commun Statist—Teory Meth*. 1998, 27(8), 1653–1675.

