# Supplementary figures and images for: Two complementary approaches to estimate an excess of mortality: The case of Switzerland 2022
Source: PLoS One. 2023 Aug 15;18(8):e0290160. doi: 10.1371/journal.pone.0290160 (PMC10426989; doi:10.1371/journal.pone.0290160)

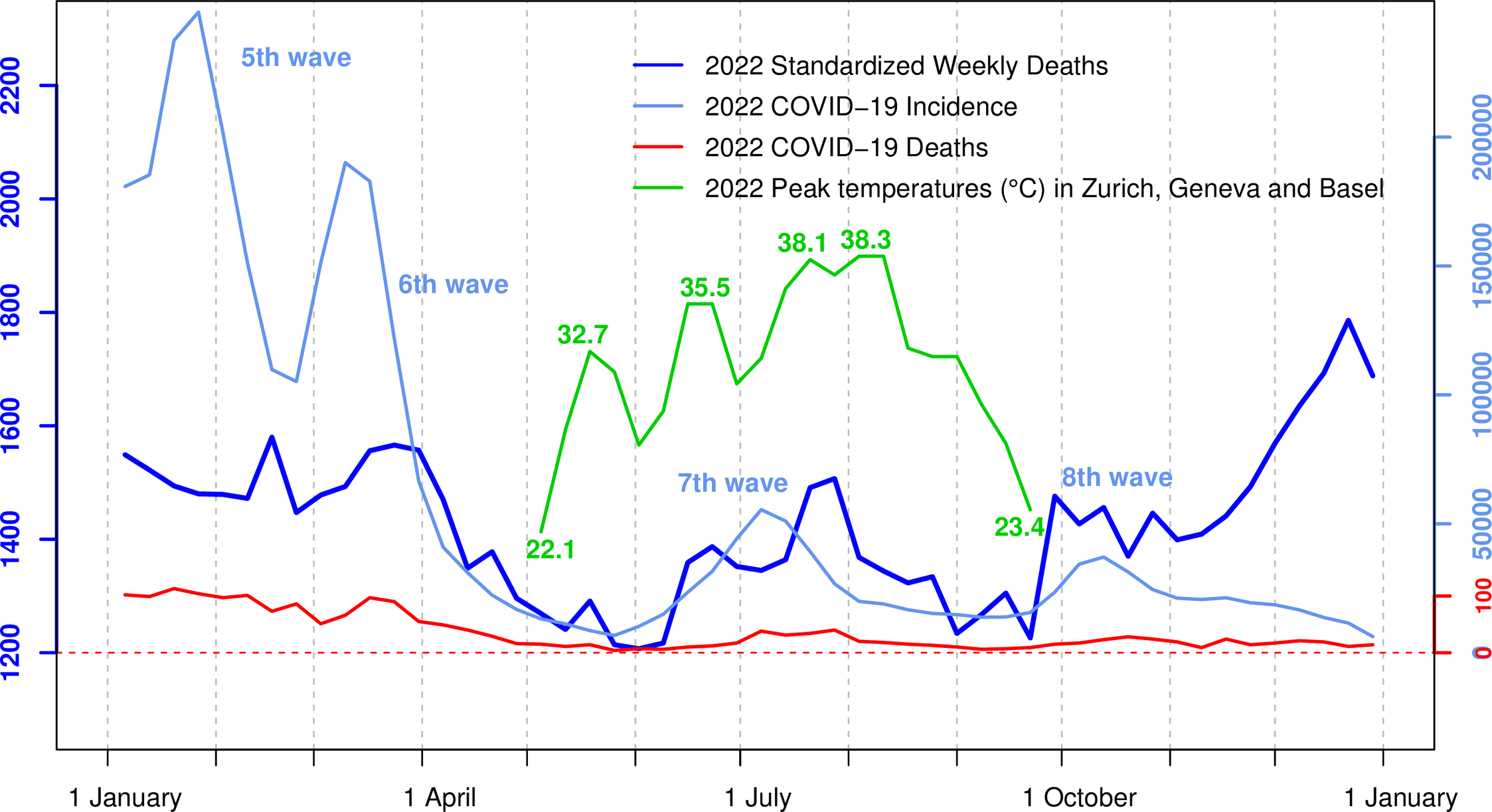

Supplement: S1 Fig — (Reference for standardization: January 1st, 2022, data from the Swiss Federal Statistical office—FSO), together with COVID-19 incidence and COVID-19 attributed deaths for the year 2022 (data from the Federal Office of Public Health—OFSP) and a temperature indicator curve giving peak temperatures among three major Swiss weather stations (data from PrevisionMeteo.ch). (TIF) [file pone.0290160.s001.tif]

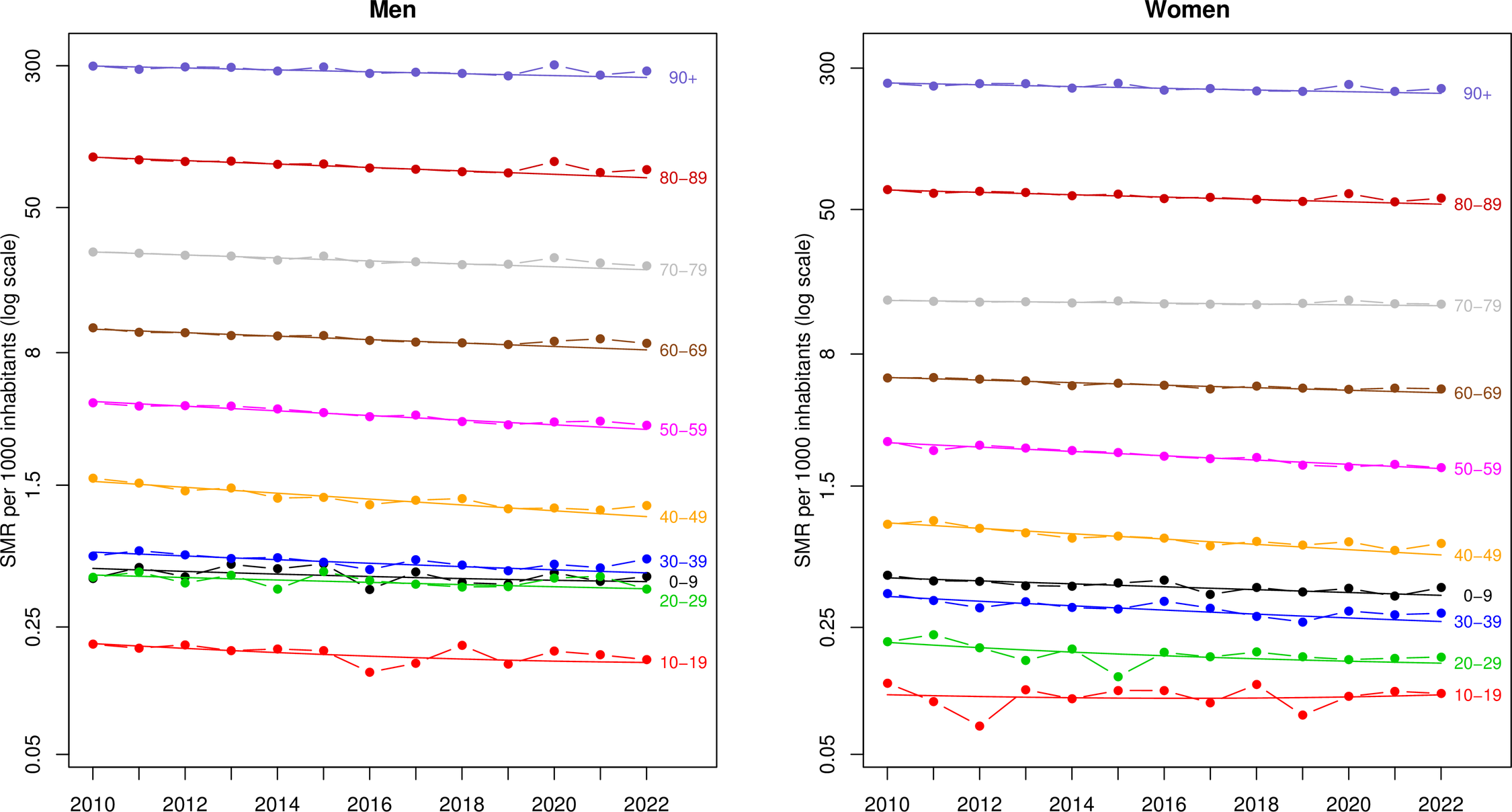

Supplement: S2 Fig — Reference for standardization: January 1st, 2022 (data from the Swiss Federal Statistical Office—FSO). (TIF) [file pone.0290160.s002.tif]
